# Paraneoplastic Pemphigus Revealed by Anti-programmed Death-1 Pembrolizumab Therapy for Cutaneous Squamous Cell Carcinoma Complicating Hidradenitis Suppurativa

**DOI:** 10.3389/fmed.2019.00249

**Published:** 2019-11-05

**Authors:** Ahmad Yatim, Gérôme Bohelay, Sabine Grootenboer-Mignot, Catherine Prost-Squarcioni, Marina Alexandre, Christelle Le Roux-Villet, Antoine Martin, Eve Maubec, Frédéric Caux

**Affiliations:** ^1^Department of Dermatology and Referral Center for Autoimmune Bullous Diseases MALIBUL, Avicenne Hospital, AP-HP, University Paris 13, Bobigny, France; ^2^Laboratory of Immunology and Referral Center for Autoimmune Bullous Diseases MALIBUL, Bichat Hospital, AP-HP, Paris, France; ^3^Department of Pathology, Avicenne Hospital, AP-HP, University Paris 13, Bobigny, France

**Keywords:** paraneoplastic pemphigus, immune checkpoints inhibitors, anti-programmed-death-1, pembrolizumab, cutaneous squamous cell carcinoma, hidradenitis suppurativa

## Abstract

A 64-year-old patient developed a widespread autoimmune mucocutaneous blistering disease 3 weeks after the initiation of the anti-programmed death-1 (anti-PD-1) pembrolizumab therapy administered for a locally advanced cutaneous squamous cell carcinoma (SCC) of the buttocks arising from hidradenitis suppurativa. A diagnosis of paraneoplastic pemphigus (PNP) was made based on the presence of a suprabasal acantholysis associated with intercellular deposits of immunoglobulin G and C3 on basement membrane zone. Analysis of the patient's sera was positive on monkey bladder and detected circulating antibodies against desmoglein 3 and desmoplakin I prior to the initiation of pembrolizumab. At that time, the patient had few localized blisters limited to the peri-tumoral skin of the buttocks with acantholysis but without *in vivo* immune deposits. Pembrolizumab therapy was discontinued and a complete remission of PNP was obtained using oral steroids. Reintroduction of pembrolizumab resulted in flare of PNP. Given the close temporal relation between pembrolizumab initiation and the subsequent clinical expression of a widespread PNP, the patient was diagnosed with pre-existing subclinical PNP exacerbated by PD-1 inhibitor. The extreme rarity of PNP in the setting of cutaneous SCC and the effects of challenge, dechallenge, and rechallenge of pembrolizumab argue in favor of a checkpoint inhibitor related adverse effect. Our case is the first PNP associated with anti-PD-1 therapy and serological follow-up suggest that one infusion of pembrolizumab is sufficient to allow clinical expression of underlying pemphigus auto-immunity.

## Background

The immune system has developed sophisticated negative regulatory mechanisms to contain the development of autoimmunity. These regulatory mechanisms can be diverted by cancer cells to limit antitumor immunity. By counteracting these inhibitory signals, cancer immunotherapy aims to enhance immune responses to fight against tumors. Among several immunotherapeutic strategies tested these recent years, immune checkpoint inhibitors have come to the forefront in cancer treatment, showing remarkable benefit in the treatment of a wide range of cancer. By blocking negative regulators of lymphocyte activation, such as cytotoxic T-lymphocyte antigen 4 (CTLA-4) and programmed cell death protein 1 (PD-1) or its ligand, programmed cell death ligand 1 (PD-L1), immune checkpoint inhibitors restore the function of effector T cells to target and destroy cancer cells. The drawback of inducing an effective immunity targeting cancers is the potential for autoimmune side effects, which are commonly termed immune-related adverse events (irAEs) (1). These manifest usually as organ-specific autoimmunity such as thyroiditis, colitis, hepatitis, or hypophysitis, among many others (2). A major challenge in the cancer immunotherapy field is therefore to increase the antitumor activity without promoting additional immune-related side effects.

Cutaneous side effects are among the most frequent checkpoint inhibitor irAEs. Skin related toxicity of any grade may be seen in up to 25% of patients treated with antibodies targeting CTLA-4 (3) and around 20% of patients undergoing anti-PD-1 treatments (4). These cover a wide range of clinical presentation such as pruritus sine materia, maculopapular eruption, vesicular or pustular lesions, alopecia, and acneiform and neutrophilic dermatosis (5). Although skin toxicity of immune checkpoint inhibitors is usually mild in severity, there are several reports of life-threatening dermatologic reactions to checkpoints blockage including drug reaction with eosinophilia and systemic symptoms (6), Stevens-Johnson syndrome (7), and toxic epidermal necrolysis (8). Moreover, several cases of autoimmune blistering diseases have been observed in patients receiving checkpoint inhibitors. So far, 34 cases of bullous pemphigoid (BP) associated with anti-PD1/PD-L1 therapy have been reported, as well as 2 cases of mucous membrane pemphigoid (MMP) (9) and 2 cases of atypical pemphigus with no mucosal involvement (10, 11). Here, we report the first case of a widespread mucocutaneous paraneoplastic pemphigus (PNP) associated with pembrolizumab therapy given for a locally advanced cutaneous Squamous Cell Carcinoma (SCC) complicating Hidradenitis Suppurativa (HS).

## Case Presentation

**Figure 1:**
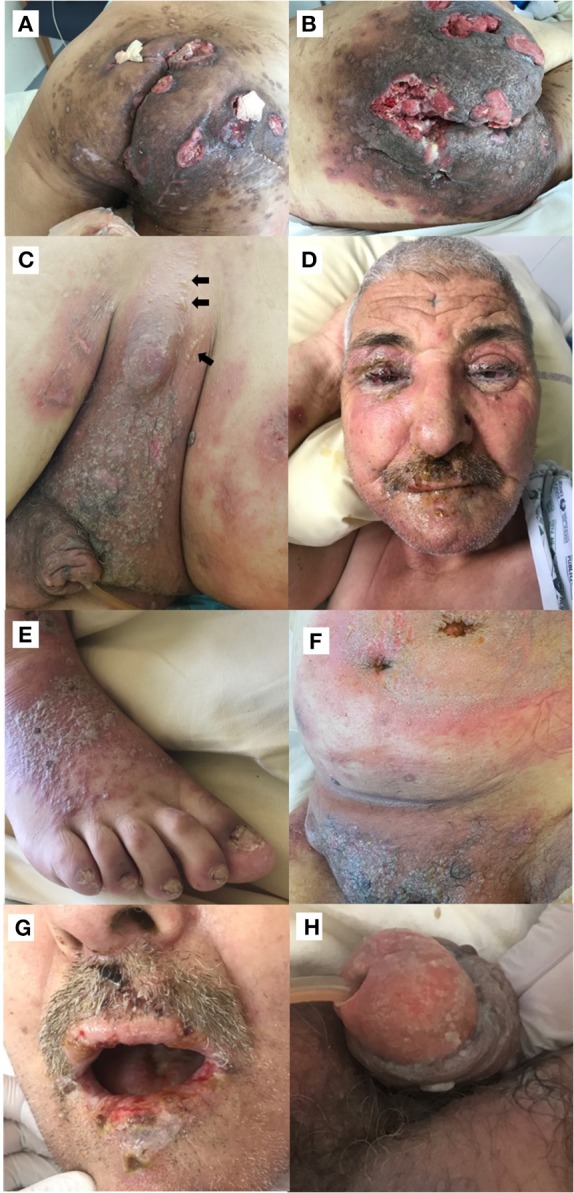
**(A)** Initial clinical presentation of the patient's buttocks showing severe hidradenitis suppurativa lesions complicated with multifocal squamous cell carcinoma. **(B)** Inflammatory swelling of the patient's buttocks and surrounding squamous cell carcinoma localization, 1 week after pembrolizumab administration. **(C–H)** Widespread mucocutaneous blistering disease 3 weeks after pembrolizumab with tense blisters (indicated by arrows) **(C)**, erythema and edema of the face, pseudomembranous conjunctivitis and eyelid erosions **(D)**, pustular lesions **(E)**, large erythematous plaques **(F)**, erosive stomatitis **(G)**, and erosions of the glans penis **(H)**.

A 64-year-old man of Algerian origin presented with a 20-year history of sinuses and abscesses of the buttocks that had been treated with several minor surgical procedures over the past 10 years. A family history was noted because the patient's son had similar early-stage lesions. Clinical examination revealed a diffuse involvement of the buttocks, perineal, and perianal regions, with multiple interconnecting tracts and abscesses across entire areas (Figure 1A). He was diagnosed with severe Hurley stage III HS. Multiple ulcerated lesions on his buttocks and large inguinal lymphadenopathies were noted. The computed tomodensitometry revealed deep tumor infiltration associated with large inguinal and external iliac lymphadenopathies, multiple abscesses of the gluteal fold, internal obturator muscles and gluteus maximus, and a sacrococcygeal osteomyelitis. Histological examination of surgical biopsies performed on skin lesions revealed moderate or well-differentiated SCC. Given the extensive local spread, the tumor was deemed inoperable.

Physical examination also revealed few small intact bullae on the buttocks and painful erosions of the buccal mucosa. There were no vesicular or bullous lesions on the upper trunk, arms, legs or extremities, and no history of a prior bullous eruption. Mouth swabs were positive for herpes simplex virus 1 (HSV-1) DNA. Histopathological examination of a buttock blister revealed intraepidermal blistering and suprabasal acantholysis. Direct immunofluorescence (DIF) was negative for deposits of immunoglobulin (Ig) G, IgA, IgM, and C3. A diagnosis of pemphigus could therefore not be confirmed. The patient was treated with oral valaciclovir, which resulted in the almost complete healing of the oral and cutaneous lesions.

Due to the poor performance status (stage 4 in ECOG scale) and high-risk of sepsis-related complications, cytotoxic chemotherapy was contraindicated for SCC treatment, and a treatment by anti-programmed cell death protein 1 (anti PD-1) was decided. One week after the first pembrolizumab administration (2 mg per kg), an intense inflammatory swelling affecting the HS areas was noted, followed by a relapse of the bullous cutaneous eruption (Figures 1B,C). Three weeks after the infusion, the patient developed erythema and edema of the face, bilateral pseudomembranous conjunctivitis with mucus discharge, and eyelid erosions (Figure 1D). Concomitantly, widespread polymorphic cutaneous lesions including flaccid and tense blisters, erosions, erythematous plaques, and pustular lesions occurred (Figures 1E,F). Mucosal examination revealed a severe stomatitis, crusting and bleeding erosions on the lips that extended beyond the vermilion border (Figure 1G), as well as erosions of the glans penis (Figure 1H). Ear, nose and throat endoscopic examination demonstrated erosions affecting the nasal mucosa, the arytenoids (75% of the surface), and the aryepiglottic folds (75%). The clinical differential diagnosis included PNP, pemphigus vulgaris (PV) and toxic epidermal necrolysis.

## Laboratory Investigations

**Figure 2:**
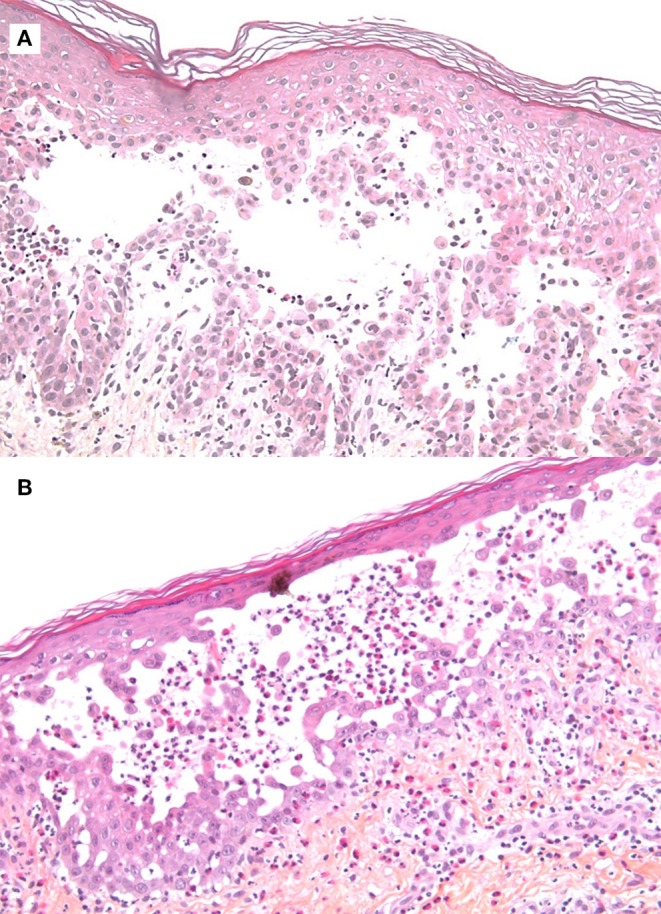
Histological examination by hematoxylin and eosin staining of skin biopsies. **(A)** Suprabasal acantholysis and intraepidermal blister formation (original magnification x100). **(B)** Intraepidermal pustule containing neutrophils and eosinophils, as well as epidermal spongiosis with exocytosis of eosinophils (original magnification x100).

Several series of skin punch biopsies were performed for histopathologic examination and DIF studies (Table 1). Histological examination by hematoxylin and eosin staining showed a suprabasal acantholysis and intraepidermal blister formations, without keratinocyte necrosis (Figure 2A), supporting the hypothesis of a pemphigus and ruling out drug-induced toxic epidermal necrolysis. A second biopsy demonstrated a spongiotic dermatitis with exocytosis of eosinophils, intraepidermal pustule formation (Figure 2B), and superficial perivascular dermal infiltrate of eosinophils and lymphocytes. Scattered necrotic keratinocytes were observed in the second biopsy. DIF of the peribullous skin revealed intercellular deposits of IgG between keratinocytes, associated with linear deposits of C3 along the basement membrane, without IgA deposits. This combined pattern (intercellular and basement membrane staining) was suggestive of PNP. Indirect immunofluorescence (IIF) testing of the patient serum on monkey bladder epithelium revealed a staining of urothelial cell surface supporting the diagnosis of PNP. Characterization of circulating autoantibodies was performed using enzyme-linked immunosorbant assay (ELISA) and immunoblotting. ELISA did not detect IgG autoantibodies to desmoglein 1, desmoglein 3, BP180 (NC16A), BP230, or envoplakin. Immunoblotting using human amniotic membrane extracts revealed a 250 kDa band corresponding to desmoplakin I but did not detect autoantibodies to periplakin (Table 1). According to the clinical presentation, the immunofluorescence pattern and the immuno-serological profile, a diagnosis of probable PNP was made.

**Table 1:** Clinical and laboratory investigations.

	**7-days before Pembrolizumab^¶^**	**20-days after Pembrolizumab**	**21-days after oral steroids^#^**
**Clinical presentation**	Small intact bullae on the buttocks, herpetic stomatitis (HSV-1 positive)	Widespread polymorphic cutaneous lesions, severe stomatitis (HSV-1 negative), erosive conjunctivitis, and glans penis erosions	Complete remission
**Histopathology**	Suprabasal acantholysis	Suprabasal acantholysis	ND
**DIF**	Negative	Intercellular deposits of IgG and basement membrane zone of C3	ND
**IIF on monkey esophagus**			
Intercellular	Positive (1/1,000)	Positive (1/400)	Positive (1/40)
Basal membrane zone	Negative	Negative	Negative
**IIF on monkey bladder**	Positive	Positive	Positive
**ELISA**			
Anti-Desmoglein 1	Negative	Negative	Negative
Anti-Desmoglein 3	Negative	Negative	Negative
Anti-BP180	Negative	Negative	Negative
Anti-BP230	Negative	Negative	Negative
Anti-Envoplakin	Negative	Negative	Negative
**Immunoblot**			
Human amniotic membrane extract	Desmoplakin I (250 kDa) desmoglein 3 (130 kDa)	Desmoplakin I (250 kDa)	Negative
Recombinant periplakin	Negative	Negative	Negative

DIF, direct immunofluorescence; IIF, indirect immunofluorescence; ELISA, enzyme-linked immunosorbant assay; ND, not done; HSV-1, Herpes simplex virus 1.

¶ *with the exception of histopathology and DIF, performed 14-days before Pembrolizumab therapy*;

# *42-days after Pembrolizumab*.

Given the presence of a localized acantholytic disease prior to immunotherapy and the short delay (<3 weeks) between pembrolizumab initiation and the widespread mucocutaneous eruption, we suspected that PD-1 blockage was associated with the aggravation of a preexisting condition, rather than the development of a *de novo* autoimmune disease. Therefore, we performed immunoblot analysis on the patient's serum collected 7 days before initiation of pembrolizumab therapy. Immunoblotting indicated the presence of circulating antibodies against the desmosomal components desmoplakin I and desmoglein 3. Consistently, IIF performed on monkey bladder epithelium using the same serum revealed a positive staining. Together, these results supported the presence of a subclinical PNP before the initiation of pembrolizumab (Table 1).

The pembrolizumab therapy was withheld and oral prednisone at 1 mg per kg daily was started. After 3 weeks of steroid treatment, complete clinical remission of the PNP was obtained and anti-desmoplakin I antibodies were no more detectable by immunoblotting (Table 1). Given the rapid progression of the SCC and the lack of alternative options, pembrolizumab therapy was restarted 6 weeks after the initial infusion while the patient remained under high dose of oral steroids (1 mg per kg daily). Pembrolizumab (2 mg per kg) resuming was associated with a PNP relapse affecting the oral cavity and nasopharyngeal mucous membrane. Immunotherapy was definitely discontinued after the third pembrolizumab dose due to grade II/III (CTCAE classification v4.0) relapsing symptoms resulting from PNP. The patient died of sepsis 3 months after initiation of corticosteroids.

## Discussion

A wide range of inflammatory skin disorders has been observed in patients treated with checkpoints inhibitors, including autoimmune blistering diseases. Current anti-PD1/PD-L1 therapy-associated autoimmune blistering diseases reported in the literature (including our patient) are presented in Table 2. To date, 34 cases of BP have been described in association with PD1 inhibitors, including 29 cases reviewed by Zumelzu (9) and five additional cases (13–16). In addition, a pharmacovigilance analysis performed on the Adverse Event Reporting System database of Food and Drug Administration recently demonstrated an increased risk to develop BP in patients treated by pembrolizumab or nivolumab (17); this signal was based on 35 case reports. Two cases of mild MMP limited to the oral cavity have also been described in patients under anti-PD1 therapy (9, 12). Apart from immune-mediated subepithelial blistering diseases, atypical suprabasal acantholytic dermatosis represents another emerging group of checkpoint inhibitor related skin toxicities. These are mainly Grover-like reactions (8 cases) and lichenoid dermatitis with suprabasal acantholysis (4 cases), without immune deposits or circulating antibodies targeting desmosomal components [reviewed in (11)]. Suprabasal acantholysis associated with immune deposits at the surface of keratinocytes has been reported in only two patients under anti-PD1 therapy. The first case reported by Ito et al. was a 68-year-old man with urothelial carcinoma treated with nivolumab who developed a polymorphic cutaneous eruption with bullae, pustules, and erosions. He had circulating autoantibodies targeting desmocollin-2 and -3, which are usually found in atypical types of pemphigus but not classical pemphigus (10). The second case was a 75-year-old man with SCC of the tongue who developed, under pembrolizumab therapy, a bullous eruption with a histopathology image and DIF pattern suggestive of PNP (11). Both cases did not show any mucosal involvement nor any other typical manifestations of PV or PNP. In contrast, our patient developed after pembrolizumab therapy a diffuse mucocutaneous eruption highly suggestive of PNP. The histopathology and serum analysis confirmed the diagnosis of PNP although immunological results were atypical by the absence of anti-envoplakine and periplakine antibodies, which are however only found in 60 and 90% of PNP cases (18). Our patient had anti-desmoplakin I antibodies which are associated with PNP in up to 47% of patients (19).

**Table 2:** Autoimmune blistering diseases associated with anti-PD1/PD-L1 therapy.

**Patients**	**Anti-PD1/PD-L1**	**Cancer type**	**Age (years)**	**Sex**	**Ipilimumab before anti-PD1**	**Cutaneous involvement**	**Mucosal involvement**	**Immunological findings**	**Delay to onset^a^ (days)**	**Rechallenge**
**BULLOUS PEMPHIGOID: 34 CASES**										
All^b^	Nivolumab: 17 (50%) Pembrolizumab: 14 (41%) Durvalumab: 2 (6%) Atezolizumab: 1 (3%)	Melanoma: 21 (62%) NSCLC: 3 (9%) Lung adenocarcinoma: 3 (9%) Lung SCC: 1 (<3%) Tongue SCC: 1 (<3%) Renal cell carcinoma: 1 (<3%) Hepatocellular carcinoma: 1 (<3%) Gastric carcinoma: 1 (<3%) Urothelial cancer: 1 (<3%) Adenocarcinoma: 1 (<3%)	Median: 70.5 (35–90)	M: 22 (68%) F: 10 (32%) Ratio F/M: 0.45	Yes: 10 (31%) No: 22 (69%) [NR: 2]	Extensive: 17 (56%) Moderate: 8 (27%) Localized: 5 (17%) [NR: 4]	Yes: 6 (19%) No: 26 (81%) [NR: 2]	DIF: positive in 28 cases (90%), negative in 3 (10%), [NR: 3]. ELISA: anti-BP180 detected in 11 out of 12 cases (92%), anti-BP230 in 1 out of 10 (10%), [NR: 20].	Median: 178 (42–588)	R0: 29 (91%) R+: 2 (6%) R–: 1 (3%)
**MUCOUS MEMBRANE PEMPHIGOID: 2 CASES**										
Patient 1 (12)	Pembrolizumab	Merkel cell carcinoma	62	M	No	No	Oral	Anti-BP180 C-terminal detected by IB	91	R0
Patient 2 (9)	Pembrolizumab	Melanoma	83	F	No	No	Oral	Lamina densa immune deposits by IEM	462	R0
**PEMPHIGUS: 3 CASES**										
Patient 1 [atypical pemphigus, (10)]	Nivolumab	Urothelial cancer	68	M	No	Moderate: Bullae, pustules and erosions	No	DIF: intercellular IgG/C3 deposits. ELISA: anti-Dsg3+, anti-Dsc2+, anti-Dsc3+. IB: negative.	188	R0
Patient 2 [atypical pemphigus, (11)]	Pembrolizumab	Tongue SCC	75	M	No	Extensive: BP-like presentation with urticarial plaques and tense blisters	No	DIF: intercellular IgG/C3 deposits and linear IgG/IgA deposits (BMZ). ELISA: anti-BP230+. IB: NR.	82	R0
Patient 3 (PNP, our case)	Pembrolizumab	Cutaneous SCC	64	M	No	Extensive: flaccid and tense blisters, erosions and pustules	Conjunctival, oral, nasal, pharyngeal, genital, anal	DIF: intercellular IgG deposits and linear C3 deposits (BMZ). ELISA: negative. IB: anti-desmoplakin I+, anti-Dsg3+	20	R+

a *Time between the first anti-PD1/PD-L1 dose and onset of the autoimmune blistering disease*.

b *All 34 cases of Bullous Pemphigoid, including 29 reviewed by Zumelzu (9) and five cases reported after 2017 (13–16)*.

*NSCLC, Non small cell lung cancer; SCC, squamous cell carcinoma; IB, immunoblot; M, male; F, female; NR, not reported; DIF, direct immunofluorescence; BMZ, basal membrane zone; ELISA, enzyme-linked immunosorbent assay; IEM, immunoelectron microscopy; Dsg3, Desmoglein-3; Dsc2, Desmocollin-2; Dsc3, Desmocollin-3; BP, bullous pemphigoid; R0, no rechallenge (no reintroduction of the treatment); R+, positive rechallenge (disease relapse after reintroduction of the treatment); R−, negative rechallenge (no disease relapse after reintroduction of the treatment)*.

PNP may have developed independently of pembrolizumab therapy. However, several data are suggestive of a checkpoint inhibitor related adverse effect in our patient: (1) PNP has never been reported previously in the setting of a cutaneous SCC, making the combination of PNP and cutaneous SCC an extremely rare phenomenon; (2) the close temporal relation between pembrolizumab initiation and the subsequent development of PNP clinical lesions (challenge); (3) the favorable outcome after pembrolizumab interruption and corticosteroids treatment (dechallenge); (4) the relapse after pembrolizumab reintroduction (rechallenge). Using the Begaud scoring system (20), we assessed the intrinsic accountability score of anti-PD1 therapy in PNP expression for our patient. With a chronological scoring (combining status of challenge, dechallenge, and rechallenge) of C3 (likely) and a symptomatological scoring of S2 (plausible), the intrinsic accountability scoring is high (I5) in our case. The two previously reported cases of pemphigus associated with anti-PD1 blockage also have high intrinsic accountability scores (I3 and I5), while <37% of induced BP reported cases were given high accountability scores (9). Although autoimmune diseases, such as inflammatory bowel diseases and spondyloarthritis, seems to occur more frequently in patients with HS, there is no association (to our knowledge) between HS and pemphigus. Interestingly, a recent article reports the development of a localized acantholytic disease associated with anti-desmoglein 1 and 3 circulating antibodies in a patient with HS of the buttocks (21). This case highlights the possibility that autoimmunity against desmosomal components might be triggered by a chronic skin inflammatory process like HS. Thus, we cannot rule out that PNP in our patient was induced by HS-related inflammation rather by the cutaneous SCC.

Several pathophysiologic models for PNP have been proposed. First, neoplasms arising from immune cell expansion have been shown to dysregulate B-cell homeostasis resulting in the subsequent production of autoantibodies targeting desmosome components. This is best illustrated by the identification of B-cell tumor clones producing antibodies targeting desmosomal plakin proteins in resected Castleman tumors (22), thymoma, and follicular dendritic cell sarcoma (23). However, this tumor antibody production hypothesis may not apply to other solid cancers associated with PNP. Alternatively, autoimmunity against keratinocytes may be the consequence of cross-reactivity between tumor antigens and normal epithelial proteins. Autoantigens routinely identified in PNP are expressed by epithelial malignancies. In our case, the patient had a skin SCC, a cancer arising from keratinocytes. Thus, antitumor immune responses to his carcinoma could cross-react with normal epidermal antigens. Therefore, distinct pathophysiologic mechanisms might explain PNP arising in the context of neoplasms involving B-cells vs. PNP associated to solid tissue epithelial tumors. Importantly, the fact that PNP is rarely associated with epithelial malignancies, which comprise the majority of human cancers, indicates that autoimmunity arising from tumor antigen cross-reactivity remains a marginal, very contained situation. This could be explained by at least two mechanisms. First, cancer cells adopt a variety of mechanisms to avoid immune recognition. Second, the immune system has evolved negative feedback mechanisms, known as checkpoints, to prevent autoimmunity. In both mechanisms, inhibitory receptors such as PD-1 and CTLA-4 have been found to play important functions (24). In our case, PD-1 blockage was rapidly associated with the expression of a severe PNP, supporting a central role of PD-1 signaling in the suppression of immune responses against antigens shared between normal and transformed keratinocytes. Because checkpoint inhibitors are now used in a growing number of human cancers, one could expect an increase in the future of cross-reactivity mediated paraneoplastic syndromes such as PNP associated to epithelial malignancies or paraneoplastic neurological syndromes triggered by the ectopic expression of neuronal antigens by tumors (25).

In summary, we reported a patient with extensive skin SCC who developed PNP after one infusion of pembrolizumab. The presence of autoantibodies directed against desmoplakin I and desmoglein 3 was demonstrated before the anti-PD-1 therapy. PNP in this patient was clearly induced since rechallenge was positive. So PNP should be added to the list of immune-related adverse events associated with checkpoint blockade such as BP.

## Data Availability Statement

The datasets generated for this study are available on request to the corresponding author.

## Ethics Statement

Written informed consent was obtained from the individual(s) for the publication of any potentially identifiable images or data included in this article.

## Author Contributions

AY, GB, and FC designed the study. SG-M conducted the immunological analyses. CP-S and AM performed the pathological studies. MA and CL collected clinical data. AY wrote the first draft of the manuscript. GB did modifications. FC and EM corrected the final version. All authors contributed to manuscript revision, read and approved the submitted version.

### Conflict of Interest

FC is a consultant for Principia Biopharma. EM is consultant for MSD and has received support for congress from BMS and MSD. The remaining authors declare that the research was conducted in the absence of any commercial or financial relationships that could be construed as a potential conflict of interest.

## Abbreviations

Anti-PD-1: anti-programmed death-1
BP: bullous pemphigoid
BP180: 180 kDa bullous pemphigoid antigen
BP230: 230 kDa bullous pemphigoid antigen
kDa: kilodalton
CTLA-4: cytotoxic T-lymphocyte antigen 4
DIF: direct immunofluorescence
DRESS: drug reaction with eosinophilia and systemic symptoms
ELISA: enzyme-linked immunosorbant assay
ENT: ear, nose and throat
HS: Hidradenitis Suppurativa
HSV-1: Herpes simplex virus 1
IgG: immunoglobulin G
IIF: indirect immunofluorescence
irAEs: immune-related adverse events
PD1: programmed cell death 1
PD-L1: programmed cell death ligand 1
PNP: paraneoplastic pemphigus
MMP: mucous membrane pemphigoid
SCC: squamous cell carcinoma.
